# Stakeholder experiences of using online spatial data visualisation tools for local public health decision support: A qualitative study

**DOI:** 10.1016/j.healthplace.2021.102648

**Published:** 2021-09

**Authors:** Roxanne Armstrong-Moore, Martin White, Thomas Burgoine

**Affiliations:** UKCRC Centre for Diet and Activity Research (CEDAR), MRC Epidemiology Unit, University of Cambridge School of Clinical Medicine, Box 285 Institute of Metabolic Science, Cambridge Biomedical Campus, Cambridge, CB2 0QQ, England, UK

**Keywords:** Health behaviours, Online spatial data visualisation tools, Acceptability, Decision-support, Qualitative methods

## Abstract

The distribution of food outlets within towns and cities and the provision of active travel infrastructure have been associated with health behaviours that can contribute to obesity risk. Decision-makers describe a lack of local data and research evidence as a barrier to policy adoption to improve the public's health. Online spatial data visualisation tools created by researchers can help to bridge this gap. We explored stakeholder experiences of using such tools for decision-support, with a focus on facilitators and barriers to use. We conducted 16 qualitative interviews with Public Health, Planning and Transport Planning professionals, who had used two recently-developed tools. Participants described the importance of tools being open access; their use in “story-telling”, particularly to non-experts; and more broadly their use even when imperfect. They expressed that ‘robustness’ of underpinning data was important, however this was not easily defined. Participants employed personal heuristics, principally based on endorsement and developer credibility, to determine suitability for use. We present key learning points for future developers to maximise engagement and impact.

## Highlights

• We conducted 16 interviews with Planners, Transport planners & Public Health professionals in England.
• These stakeholders were users of online spatial data visualisation tools, who deemed them acceptable.
• Robustness of underpinning data was an important precursor to use but was not easily defined.
• Participants assessed developer endorsement and credibility as a marker of quality, which affected use.
• Barriers and facilitators to use should be considered by developers to increase engagement & impact.

## 1 Introduction

Obesity is a major risk factor for many non-communicable diseases across the world, including type 2 diabetes and some cancers (World Health Organization, 2018). In 2017, the Health Survey for England reported that 64.3% of adults were overweight or obese (NHS Digital, 2017), with predictions that levels of obesity will continue to rise both here and across the world (Di Cesare et al., 2016). Moreover, the burden of obesity across the UK population and elsewhere, is greatest among disadvantaged groups (Loring and Robertson, 2014).

The determinants of physical inactivity and poor diet, which can lead to obesity, are many and varied. The distribution of food outlets in our towns and cities, and other aspects of urban design such as the provision of infrastructure for active travel, have been associated with these health behaviours (Kärmeniemi et al., 2018). For example, greater exposure to fast-food outlets within residential neighbourhoods has been associated with poorer diet and greater obesity risk (Burgoine et al., 2018). Moreover, evidence shows that deprived areas have a higher density of fast-food outlets (Public Health England, 2018), contributing to observed social inequalities in health (Burgoine et al., 2018). Elsewhere, research has demonstrated that access to higher quality active transport infrastructure contributes to an increased likelihood of walking and cycling (Aldred et al., 2020; Panter et al., 2016).

Despite growing evidence for these upstream determinants of health, to address high levels of obesity governments have historically focussed on targeted public health interventions and/or those that draw heavily on individual agency, such as improving nutrition education in schools or front of pack nutrition labelling (Adams et al., 2016). Such approaches have failed to curb population levels of obesity, especially in children (Waters et al., 2011), and may widen existing health inequalities (White et al., 2009). Public health interventions that shape the built environment to promote healthy behaviours, thereby targeting whole populations, have become more common and are likely to be more effective and equitable at a population level (Adams et al., 2016; McGill et al., 2015). Examples include the provision of higher quality or more complete walking and cycling infrastructure such as footpaths and cycle lanes, or restrictions placed on new fast-food retailing around schools using the urban planning system.

In England and elsewhere, place-based interventions are often implemented by local authorities (LAs) within local government areas. In this context, policymakers use local data to support their decision-making, and have described a lack of local data as a significant barrier to policy adoption (Oliver et al., 2014; Oliver and De Vocht, 2017; Orton et al., 2011). Such data include, for example, information regarding the spatial distribution of risk factors for obesity in the built environment. This data is accessed increasingly by a diverse “neogeographical” user base (Byrne and Pickard, 2016), including those who historically might have been unfamiliar or less skilled with spatial data and its applications, through online spatial data visualisation tools such as SHAPE (www.shapeatlas.net). These tools typically provide data in an accessible and interactive map-based (i.e. spatial) format, as distinct from, but which might also include other forms of (aspatial) data visualisation such as graphs and charts (Monsivais et al., 2018).

Until recently, there have been relatively few attempts made by the research community to share public health research evidence and spatial data via online tools. However, researchers have begun to exploit a growing familiarity with such tools to both effectively share data from and actively engage users in research projects. Moreover, this practice is incentivised as a new form of “pathway to impact”, which is a UK research funding priority (Blagden, 2019; Watermeyer and Chubb, 2019). Yet there is limited evidence regarding the acceptability of online spatial data visualisation tools that have been *developed by* researchers, and the barriers and facilitators faced by policy makers, which might affect uptake.

Examples of researcher-led online spatial data visualisation tools include the Food environment assessment tool (Feat, www.feat-tool.org.uk) and the Propensity to Cycle Tool (PCT, www.pct.bike). Feat is an online tool that allows users to map, measure and monitor, regional and neighbourhood exposure to six types of food outlet across Great Britain, including over time (Monsivais et al., 2018). Feat was designed for an audience of planning and public health colleagues in local authorities. PCT is an online tool that shows baseline cycling levels and scenario-based cycling potential across the UK (Goodman et al., 2019; Lovelace et al., 2017a, Lovelace et al., 2017b), which was designed to assist transport planners in prioritisation of investment in cycling infrastructure. Further details of both tools have been published previously (Lovelace et al., 2017a, Lovelace et al., 2017b; Monsivais et al., 2018). Other researcher-led spatial data visualisation tools permit, for example, mapping of alcohol and tobacco outlets in Scotland (creshmap.com), of small area deprivation estimates across England (maps.cdrc.ac.uk), or modelling of flood risk e.g. KEEPER (Alexander et al., 2013).

The aims of this study, therefore, were to understand the acceptability of online spatial data visualisation tools developed by researchers, to policymakers, including planners and those responsible for public health, as well as facilitators of and barriers to their use.

## 2 Methods

### 2.1 Design

We used semi-structured telephone interviews to address our aims. In conversation with Feat and PCT users, we explored use of these specific tools, which were both developed by University of Cambridge researchers, as vehicles or ‘jumping off points’ through which to generate wider learning about online spatial data visualisation tools.

### 2.2 Participants and recruitment

Purposive sampling was used, drawing on a sampling framework to ensure representation across key user groups working within or on behalf of local authorities: individuals from local authority Public Health, Planning and Transport Planning departments; Transport Consultancies working on behalf of local government; and regional Public Health England (PHE), which is an executive agency of the Department of Health and Social Care with responsibility for advising local authorities on matters including public health. We planned to recruit up to a maximum of 25 participants, which we felt based on prior research experience would be sufficient enough that no new data would start to be provided in interviews i.e. that data saturation had been reached (Fusch and Ness, 2015).

Various recruitment strategies were employed. We contacted individuals who had previously tested these tools during development; those who had attended tool training sessions; those who had emailed the tool developers requesting more information or assistance; and those who were otherwise known to the developers as users. All individuals had previously expressed interest in or consented to being contacted for the purposes of subsequent research. Individuals were approached via email and provided with an information sheet that explained the aims of the study. They were asked if they would be happy to take part in an interview regarding their use of Feat or PCT within the decision-making process. After first contact, individuals were followed up twice over two weeks. Non-responders were not subsequently contacted.

### 2.3 Materials and data collection

All study materials were approved by the University of Cambridge Humanities and Social Sciences Research Ethics Committee (Reference: 19/192). These included an interview topic guide that we developed to help address our aims (see appendix 1), based on a previously published theoretical acceptability framework (Sekhon et al., 2017). The topic guide covered: use of geo-spatial tools and examples of use; use of Feat or PCT specifically, and examples of use; and barriers and facilitators to using geo-spatial data tools with a focus on Feat or PCT. The interviews were semi-structured, and the topic guide was used flexibly, giving participants the opportunity to add any information they felt would be useful and allowing the researcher leading the interviews to gain more information if required. Field notes were taken by the researcher throughout each interview to highlight any emergent findings. Each interview ended with a recap of points discussed, which gave participants the opportunity to confirm these, ask questions and make any additional comments they felt had been missed or to provide clarification.

Interviews were carried out between March 2019 and January 2020. The participants were made aware that the interviewer (RAM) was not an expert in either tool, and had played no role in the development of either platform. This was to reassure the participants of the interviewer's neutrality and position as a “learner” in this context (Blaikie, 2009), and encourage openness and self-expression in the answers given. Participants were informed that the interview would take around 45 minutes. Interviews were recorded using a digital audio recorder, transcribed verbatim and anonymised by a professional transcription agency. All data were held according to standard MRC Epidemiology Unit Data Management and storage protocols, compliant with UK law and national standards for research governance.

### 2.4 Data analysis

Data were analysed using the Framework approach, which was developed as a data analysis tool specifically for policy research (Ritchie and Spencer, 1994). The constant comparative method was used throughout, which involves making comparisons between concepts and ideas during each stage of the analysis, allowing the data to be enriched as it is collected.

Tool developers were involved in the analysis of data for the purposes of aiding understanding and facilitating interpretation. All analyses were cross-checked and agreed upon by all members of the research team to minimise risk of bias, and RAM maintained complete ownership of the analysis. RAM, TB and MW (alongside project collaborators JW and RA) independently coded 10% of transcripts and created an initial framework based on these codes. This framework was applied to the remaining transcripts, but was used flexibly, allowing for new codes to be added where necessary. Codes were subsequently grouped into themes, which aimed to best describe the data. To aid analysis, transcripts were imported into computer aided qualitative data analysis software NVivo 12 (QSR International Pty Ltd, 2020).

## 3 Results

We completed interviews with 16 participants from key user groups: six individuals from Public Health and one from Planning from across six local authorities, four from Transport Planning across a further four local authorities, one from regional Public Health England, and four from three different Transport Consultancies (Table 1). Two participants belonged to the same local authority but were recruited from different departments. Two participants belonged to the same department within one transport consultancy.

**Table 1:** Characteristics of study participants (n = 16).

Participant ID^a^	Organisation	Role	Tool used
LA01	Local Authority	Public Health	Feat
LA02	Local Authority	Public Health	Feat
LA03	Local Authority	Public Health	Feat
LA04	Local Authority	Public Health	Feat
LA05	Local Authority	Public Health	Feat
LA06	Local Authority	Public Health	Feat
PHE01	Regional PHE	Public Health	Feat
LA07	Local Authority	Planning	Feat
LA08	Local Authority	Transport Planning	PCT
LA09	Local Authority	Transport Planning	PCT
LA10	Local Authority	Transport Planning	PCT
LA11	Local Authority	Transport Planning	PCT
CO1	Consultancy	Transport Planning	PCT
CO2	Consultancy	Transport Planning	PCT
CO3	Consultancy	Transport Planning	PCT
CO4	Consultancy	Transport Planning	PCT

a Quotations in the Results are labelled as LA (local authority), PHE (regional Public Health England) and C (consultancy).

Five themes were derived from our analysis of the data: making data accessible online; use of imperfect but best available data visualisation tools; credibility and endorsement of spatial data visualisation tools; robustness of data; and technical barriers to use and application. These themes are described in detail below, with quotations provided to illustrate concepts.

### 3.1 Making data accessible online

Users reported that having data presented in map form aided their interpretation of inherently spatial data, helping them to make sense of information that would be less interpretable in other forms (e.g., spread sheets, graphs). Further, while individuals in this study stressed that everyone interprets data differently, spatial data visualisation tools were perceived as making data especially accessible to non-specialist audiences such as elected members of local government.

*"The ability to visualise it [the data] on a geospatial view as opposed to just seeing it on a spreadsheet or in a graph. It gives you another dimension…"*

C04 (Transport Planning)

Spatial data visualisation tools allowed individuals and organisations to understand important societal and public health issues. Specifically, the ability to visualise data helped reaffirm existing organisational priorities. At the same time, the tools also highlighted new areas of focus, including geographic disparities between areas that users were not necessarily aware of, which may have implications for strategic policymaking.

*"And it [ability to represent data visually] sort of backs up to the reasoning why you're doing something, why you're requested funding, because then someone can see exactly what it is you're trying to do, what the connection is that you're trying to make."*

LA08 (Transport Planning)

*"It's almost like it's opened up a whole new area that we weren't really, wasn't really part of our consciousness before."*

LA02 (Public Health)

Individuals commented that the spatial data visualisation tools discussed were straightforward to use. They stated that this is a must when using any tool, as aside from their own use, the ability to share tools with other individuals and across departments and disciplines, without the need for further explanation, was a facilitator of use, ultimately saving both them and their organisation time.

*"I think it's a very easy to use application compared to [other] similar ways of trying to get hold of that data […]. But, yeah, I think it produces [a] very easy to use representation of the data and [there's a] really simple way to download the background data as well behind it."*

LA11 (Transport Planning)

*"I think any of my colleagues both in public health and wider would be able to go in there and use it straightaway. If they couldn't I'd be questioning their IT skills, let's put it that way."*

LA06 (Public Health)

Individuals described how these data visualisation tools gave them access to ‘off the shelf’ processed data in a ready to use format, which served as a facilitator of use. Analysing raw data would be more onerous, if indeed it were even possible.

*"… it's easier to get hold of that rather than me having to try and analyse it using sort of MapInfo […] you know it's easier to just access it so in that respect it makes that data easier to access."*

LA08 (Transport Planning)

The tools discussed in this study were available publicly online and at no cost, which served as facilitators of use. Individuals reported the benefits of online access, permitting continuity of established ways of working, even as they moved between departments and organisations.

*"I actually came to a meeting [about the tool] when I was in my previous role in [another local authority], so I've actually been using [the spatial data visualisation tool] when I was working [there] as well […] I just brought it with me from using it [there] really, and I started using it from going to this meeting, and learning about the tool, and how it worked."*

LA02 (Public Health)

The limited financial resources typically possessed by local authorities also meant that the tools being free to use served as a facilitator of use.

*"I think as a team our, you know, kind of like resources are quite stretched so having that tool that was pretty much easy for anyone to use was a real benefit."*

LA02 (Public Health)

Participants indicated that they used spatial data visualisation tools to create outputs to show to policy makers and clients. These outputs could be shared quickly and easily, aided by online accessibility at no cost.

*"Being able to kind of show them a snapshot of all the work you've done […] they can grasp it within five minutes, it's very valuable for the client … it saves time."*

C01 (Transport Planning)

However, there was awareness among those interviewed that the financial situation of local authorities is very different across the country, and that this might influence uptake. Greater London was described as being potentially better resourced than other regions.

### 3.2 Use of imperfect but best available data visualisation tools

Users need access to data visualisation tools that are fit for purpose. Sometimes, tools used by participants did not offer the exact functions required, such as the ability to layer or add their own data. Individuals suggested that the tools they had access to were used because there was no alternative.

*"So in local government you, probably not, less than academia, you take a practical course rather than necessarily the precise course, a kind of good enough question, data collection, data analysis."*

LA09 (Transport Planning)

While not ideal, this was not reported as a barrier to use, with participants still citing use of existing tools as the best available option.

*"I don't think there's an alternative out there. Unless you're creating your own GIS style with data from the local authority, or data that you have."*

C01 (Transport Planning)

*"It's not like we're all told that we have to use [a given] tool but I suppose it's like the best thing that we've got."*

LA08 (Transport Planning)

There was a real emphasis in this study on ‘progress over perfection’, and that using a tool that provided access to some data was better than having no data at all.

*"We're quite sort of grateful to see these new types of tools developed because they save us time basically and make it easier to synthesise that kind of information."*

LA02 (Public Health)

### 3.3 Credibility and endorsement of spatial data visualisation tools

Use of a spatial data visualisation tool was often based on an individual's judgement of the *credibility* of the developers, and whether the tool had received any *endorsement* by trusted external bodies.

#### 3.3.1 Credibility of the developer

Credibility of the tool developer was deemed important by individuals in this study. When tools were developed by an organisation they deemed to be “credible”, individuals felt more confident with its use. Credible organisations were commonly described to be academic institutions.

*"Is it a credible organisation, is it good universities, is it a national source, international source, that's another thing we will look at as well."*

LA07 (Planning)

On the other hand, there was a perception that a tool coming from a commercial company might involve conflicts of interest. This would result in a lack of trust and would lead individuals to interpret data emerging from such tools with more caution.

*"I guess if it came anyone with like kind of ulterior motives, that might be, as if the [popular motoring association] decided they were going to publish some details on walking and cycling we'd probably be less inclined to look at it."*

CO3 (Transport Planning)

#### 3.3.2 Endorsement

Endorsement, which participants described as a hallmark of data quality, and conferred by an establishment such as the Department for Transport, the Faculty of Public Health or Public Health England, facilitated confident use of spatial data visualisation tools. Endorsement was especially important when results were being presenting to others beyond their organisation, or to colleagues in other departments.

*"Endorsement means it has got that validity to it, that it has been checked out, that it meets those standards of data validation or research validation."*

PHE01 (Regional PHE)

*"[Is] it endorsed, has it been used by someone else, has it been acknowledged by someone else, or by somebody who is good in that field or experts in the field[?]. Because if Public Health have endorsed it, or if they say, you know, it's a good data, then obviously we will look into it, or we will use it."*

LA07 (Planning)

If a tool was fit for purpose but was recommended by a body that was not widely approved of, it was tentatively reported that this tool would still be used, but information regarding the source would not be made public.

*"I mean, if, you know, if a tool was being put forward by a body that one disapproved of or disagreed with … but you still wanted to make use of the tool, then I suppose you'd just simply not mention that."*

LA11 (Transport Planning)

Throughout the interviews, the actions of other local authorities were highlighted as important, and with respect to tool use, as a form of endorsement that might facilitate uptake. There was also a sense that if other areas were doing well and hitting targets, lessons might be learned in terms of how this success was being achieved, which might include the use of tools.

*"I think you can take inspiration from other people and you do … you look at what they've done, and try and use the same tools."*

LA01 (Public Health)

### 3.4 Robustness of data

Participants showed a strong awareness of the importance of the robustness of underpinning data, and the impact of subsequent data processing, which might affect whether the data would ultimately “stand up in court” if challenged.

*"But, yeah, the source does matter, because ultimately if someone comes back to use and says where's this data come from, you need to be able to say and it needs to be reliable."*

CO3 (Transport Planner)

*"So we make sure that a) it's robust enough and b) it will, you know, stand in the courts if ever we are challenged on the use of the data."*

LA07 (Planning)

As “public money” funds both users of tools in local authorities and subsequent public health decision-making, this heightened awareness of the need for data and its empirical underpinnings, to be robust.

*"When you're using public money to try and push some of these then you know, the evidence based needs to be pretty robust."*

C02 (Transport Planner)

The provenance of underpinning data, including considerations such as where the data were sourced from, and clarity as to how the data were processed, were described as key factors in determining robustness and therefore use.

*"Everyone wants to know how, how this data has been acquired and how […] the tool has come to this conclusion of where these routes are."*

CO1 (Transport Planning)

To some extent, assessments of robustness were also tied to credibility of the developer. If the tool was developed by an academic institution, particularly one that individuals considered to be “prestigious”, this would be taken as a mark of data quality and checks on robustness might be less rigorous.

*"I think being produced by [prestigious university] it does come with a kind of quality mark, you know, there's names on respected academics, published papers, you kind of just know that you're looking at something that's been produced by people that know what they're talking about."*

LA03 (Public Health)

A commercially produced tool would be less trusted, for example from a developer with a perceived conflict of interest. Individuals were likely to investigate this data more, potentially choosing not to use it as a result.

*"I think the fact that it’s an education institute gives it a lot more credence than if it was, I don't know, [name of popular restaurant chain] created their own data source, so yeah, it is important that it comes from a recognised institution that's known for the work that they do."*

LA01 (Public Health)

However, definitions of what constitutes “robust” varied. Despite an emphasis on data quality, it was apparent that there was no gold standard method for assessing this and the true extent of data checking appeared variable. Individuals used their own methods for checking data, which included assessment of face validity using local intelligence and data.

*"We will have our local knowledge, intelligence people who are all the people that are used to handling any kind of data or whatever and therefore can apply all those kind of tests as to the robustness of data from a statistical, technical point of view."*

PHE01 (Regional PHE)

Overall, individuals seemed to employ a personal heuristic when assessing quality of data, using developer credibility, knowledge of the data source and evidence of endorsement to make this judgement.

*"I think because [name of data visualisation tool] is within guidance, it's within kind of Department for Transport guidance, so it's kind of seen as valid enough."*

C01 (Transport Planning)

*"It probably would to some extent, again it comes back to kind of trust, I think the more people that you know that are using something, the more trust that you tend to have in it, you know somebody else who's also kind of quality checking it."*

LA03 (Public Health)

### 3.5 Technical barriers to use and application

In terms of barriers to use, some individuals expressed concerns related to perceived technical inability, lack of expertise and the potential need for training to exploit the advanced functionality of these tools.

*"Maybe just lack of knowledge about what it is and how to use it. When you first turn it on and you get those kind of spikey maps that come out it can seem a bit daunting like it's not really clear what's going on and people need a bit of training basically."*

LA10 (Transport Planning)

Where individuals needed support with tools, they reported this being easy to access. They appreciated having training opportunities and support in person or via email from developers.

*"I mean I have to say you know credit to the people that developed this one […] but it was well-explained to us and you know both P and T were very helpful in terms of dealing with comments and queries that people had both at the presentation and afterwards as well."*

PHE01 (Regional PHE)

Computing infrastructure within local authorities, such as internet bandwidth and restrictions placed on web-browsers, were also described as a barrier to using these tools effectively.

*"It just needs more server bandwidth […] like all things do, sometimes it's a bit slow to respond […] when you change something you're changing the data that you're seeing and it can be quite laggy."*

LA10 (Transport Planning)

*"Yes I think it is or I just think limit, you know with the IT systems of like local authorities it just, I don't know just didn't want to do it for whatever reason. Some of it was sort of compatibility … when I've been to some of the workshops they've done on the [name of data visualisation tool] they tend to use a different system so it sort of seems more compatible [and] easily to use it on that."*

LA08 (Transport Planning)

Individuals described the possibility for organisational inertia i.e. resistance to change within organisations when determining whether to use a new tool, to exist. However, they were confident that with management and education, and clear evidence of usefulness, this would be overcome. Moreover, individuals expressed that use of a new tool is often an individual, as opposed to an organisational-level decision.

*"I can use whatever tools I want, I take advice, but yeah, if I'm happy using a tool then I don't really have any interference from anybody."*

LA01 (Public Health)

## 4 Discussion

### 4.1 Summary of findings

This is the first study to determine the perspectives of those working within or on behalf of local government in England, regarding the acceptability of, barriers to and facilitators of using spatial data visualisation tools *developed by* researchers to support local decision making. Aided by online availability at no cost to the user, our findings suggest that online spatial data visualisation tools are an acceptable vehicle for communicating effectively with a range of audiences, and non-experts in particular, where they can be used to aid “story-telling”. Participants suggested that whilst the tools discussed were not perfect, they were the best they had available and this did not preclude use. Importantly, the decision to use a tool was based on a combination of developer credibility and endorsement, conferred by external bodies and use elsewhere. ‘Robustness’ of data was also a facilitator of use; however, this was hard for participants to define. Technical barriers, including computing infrastructure within local authorities, were perceived as a potential barrier for effective use. The decision to use a tool was described as being made by individuals not organisations.

### 4.2 Comparison to previous research

The findings of this analysis are similar to those of previous research regarding the use of academic outputs and data in policymaking. The importance of transparency in the reporting of methods, combined with accurate and robust information, remains critical to achieving engagement and impact (Whitty, 2015). Our participants reported applying a personal heuristic when evaluating a tool for use. They primarily considered factors such as the origin of information and credibility of developers as indicators of validity, reliability and robustness. But similar to Sekhon et al. (2017), we also found that the attitudes of others can impact acceptability. Specifically, use of a tool by others was seen as an additional form of endorsement that played into decision-making surrounding use. Whilst it was possible that organisational inertia might inhibit the adoption of new tools, and in particular those emerging externally e.g. from academic developers, we found that the decision to use a tool was made at the individual, as opposed to organisation level. This autonomy allows for agile and responsive users.

Previous research has shown the importance of local data for decision-making, with policymakers valuing data that reports on the needs and behaviours of the local population (Oliver and De Vocht, 2017). Our observations corroborate these earlier findings, but in the specific context of where data is accessed from a researcher-led online spatial data visualisation tool. Importantly, participants reported how data were used both to reaffirm existing organisational priorities, as well as to highlight new areas of focus, acting as reported elsewhere as conversation starters, able to underpin new ways of working (Feat Development Team, 2020). This speaks to the ability of data visualisation to communicate not only what is known, and to help users explore and visualise their existing concerns, but also to discover what is *not* known and to spark creativity (Alexander et al., 2013; MacEachren, 2001). The latter a result of a user or users (Feat Development Team, 2020), bringing to bear their local knowledge in the construction of bespoke outputs that blur the boundary between knowledge producer and knowledge user (Alexander et al., 2013).

Moreover, local government funding, specifically for public health functions has been reduced over recent years, while at the same time, in 2013 the responsibility for delivering such services was moved from the NHS to local government (Local Government Association, 2018a). As a result, individuals in this study indicated that their organisations were often stretched in terms of time and financial resources, and therefore they were happy to use open access and free tools as a source of local data, and that they were grateful for the existence of these tools even if they did not entirely fit their needs.

### 4.3 Implications for developers

To the extent that our data allow it, we have tried here to summarise the implications of our findings for researchers developing their own online spatial data visualisation tools.

It was clear that policymakers placed a strong emphasis on tools having emerged from high quality research, underpinned by robust data, and that this was a key determinant of use. From a legal perspective this was especially important i.e. users need to be confident that the data would “stand up in court” if called upon to do so. Developers can be proactive in demonstrating these important attributes through clear documentation of data sources and methods, including links to evidence (such as peer-reviewed journal articles) that substantiate veracity. Tool endorsement was also important and was frequently taken together alongside developer credibility to inform opinion regarding the likely quality of underpinning data. Some forms of endorsement, such as from a professional membership organisation or charity, can be actively sought out by developers. Other forms of endorsement, such as use by an influential individual or organisation, which might be expected to generate a ripple effect in subsequent use (especially given the agility of the audience), may also be strategically important. Related to credibility, it was also critical that the individual or organisational sources of data were free from conflicts of interest. Researchers should try to avoid perceptions of these conflicts from arising.

Cairney and Oliver (2017) have previously suggested that academics should communicate well with their audiences, allowing their research to be relevant and readable and to bridge the gap between evidence and policy. Accordingly, ease of use and “approachability” were reported as paramount, suggesting that developers should focus on maximising these to drive user engagement, and to empower (not exclude) non-GIS expert users who are perhaps engaging with unfamiliar geographical concepts and techniques (Haklay et al., 2008). More broadly, this study suggests that online spatial data visualisation tools can act as an acceptable vehicle for communicating effectively with a range of different audiences. In part, this was due to the power of data in map form to tell a ‘story’ (Davidson, 2017), and to communicate relationships between data that may not clear through other modes of data presentation (Nykiforuk and Flaman, 2011). Participants within this study believed this to be especially true for a lay audience such as elected members of local government, who play a critical role in decision making. As well as being non-specialists, elected members are often stretched across myriad different issues (Local Government Association, 2018b), therefore having access to data that is easy to interpret is key.

Part of communicating well is also to develop a tool that is relevant and suited to its intended application and, to ensure this, developers should work *with* and learn from potential users. That said, a key finding of this research was the value of an imperfect tool, and the flexibility demonstrated by users to work effectively with the data at their disposal. To some extent it is also true that “the novelty of any tool is likely to be part of its appeal” (Monsivais et al., 2018). While developers should certainly attempt to understand their “customer”, the development of something, even if imperfect, was clearly seen as preferable to having nothing at all. An imperfect tool can also be viewed as a pilot, from which to obtain feedback and evolve.

Being accessible to policymakers through active engagement builds trust and facilitates uptake (Oliver et al., 2014). Individuals in this study emphasised the importance of being able to communicate with individuals from a tool's development team, be it via email or during training sessions, to ask questions or voice concerns, such that the developer can become a ‘trusted voice’ (Oliver and Cairney, 2019). Active engagement also involves a thoughtful dissemination and engagement strategy (Oliver and Cairney, 2019), with users noting the particular importance of meeting developers. Monsivais et al. (2018) suggest a range of possibilities for engagement, including promotion through social and news media, face-to-face meetings, and workshops and other outreach events, with a focus on non-academic audiences. It may be necessary to employ a dedicated ‘knowledge broker’ to aid with this process (Marshall and Cvitanovic, 2017; Quarmby, 2018).

### 4.4 Strengths and limitations

This is the first study to investigate views of individuals working in local authorities and transport consultancies, regarding their use of online spatial data visualisation tools. In our position as recent developers of two such tools, we were uniquely positioned to exploit our contacts with users in order to permit this research. We used qualitative research methods to explore a detailed and rich account of the views of these individuals. In turn, this allowed us to make recommendations for prospective developers of online spatial data visualisation tools.

These strengths are balanced by a number of limitations. Within the public health sector there is an emphasis placed on evidence that is “systematically generated and analysed, with interpretations that are well founded and defensible and able to support wider inference” (Ritchie et al., 2013, p.21). However, the findings of our qualitative research may not be generalizable to all users. For example, our interviews were focussed on use of Feat and PCT, as talking points from which to develop more generalised learning. It is possible that our findings are specific to users of only these tools, and it was not feasible to include users of other tools, which may have yielded different opinions, in this analysis, However, Feat and PCT are in themselves different tools with their own unique functions upon which participants could reflect, and each have their own user groups, across which we recruited in order to ensure a diverse range of voices were heard. While no further interviews were conducted when it was deemed that interviewees had ceased offering new insights, it is possible that further interviews might have yielded new information. It is also possible, although we applied our topic guide flexibility to accommodate the responses of interviewees, that the data generated were a function of the questions asked. These are potential limitations of all qualitative research. We were unable to identify ‘non-users’ of these tools e.g. those who had tested a tool and ultimately decided not to continue its use. These individuals are likely to hold a unique, critical perspective regarding online spatial data visualisation tools, and could be the focus of future research. While we took steps as described to minimise bias that could have occurred as a result of participants being contacted by a researcher from the university at which the tools were developed, we cannot eliminate this possibility. Future studies undertaken by a researcher with no institutional affiliation to that of the developer, would further minimise the possibility of bias.

## 5 Conclusions

Based on a discussion of the use of two researcher-led online spatial data visualisation tools (Feat and PCT), individuals working within and on behalf of local government were broadly accepting of these tools as vehicles for effective communication of local data. They emphasised the importance of these tools being open access and free at the point of use, and their ability to tell a story to which other forms of data presentation are less suited, especially to non-experts. They expressed that ‘robustness’ of underpinning data was important, however this was not easily defined. This frequently resulted in individuals employing personal heuristics, principally based on endorsement and developer credibility, to determine suitability for use. Facilitators and barriers to the use of online spatial data visualisation tools as identified here, might usefully guide the future development of other such tools, in order to maximise engagement and impact.
