# Supplementary material for: Stakeholder experiences of using online spatial data visualisation tools for local public health decision support: A qualitative study
Source: Health Place. 2021 Sep;71:102648. doi: 10.1016/j.healthplace.2021.102648 (PMC8520943; doi:10.1016/j.healthplace.2021.102648)
Supplement: Multimedia component 1 [file mmc1.docx]

| Can you tell me about your current role?  Can you tell us about the priorities of your organisation and how they inform your work?  What are the potential barriers to achieving these priorities?  How important is local geospatial data to you and your organisation?  Now I’d like you to give me one example of local geospatial data (not Feat/PCT) and tell me a bit about how you use it or have used it in the past.  Moving onto Feat/PCT, I’d now like to ask more detail about your use of Feat/PCT within your current role.  Can you remember how you first heard about Feat/PCT?  Can you tell me how you use Feat/PCT?  How does Feat/PCT fit within the decision-making process (e.g., urban/transport planning)?  What kinds of local authorities have commissioned work on PCT? (For consultancies only/stakeholders with national overview).  Are there some local authorities who are less interested in PCT? (For consultancies only/stakeholders with national overview). |
| --- |

Appendix 1. Final interview guide
